# COVID-19 myocarditis: a case report

**DOI:** 10.31744/einstein_journal/2020RC5876

**Published:** 2020-10-20

**Authors:** Patrícia Yokoo, Eduardo Kaiser Ururahy Nunes Fonseca, Roberto Sasdelli Neto, Walther Yoshiharu Ishikawa, Murilo Marques Almeida Silva, Elaine Yanata, Rodrigo Caruso Chate, Antonio Carlos Bacelar Nunes, Marcelo Bettega, João Ricardo Cordeiro Fernandes, Flávio Tarasoutchi, Gilberto Szarf

**Affiliations:** 1 Hospital Israelita Albert Einstein São PauloSP Brazil Hospital Israelita Albert Einstein , São Paulo , SP , Brazil .

**Keywords:** Coronavirus, COVID-19, Coronavirus infections, Myocarditis, Cardiomyopathies, Communicable diseases, Severe acute respiratory syndrome, Pneumonia, Echocardiography, Tomography, X-ray computed

## Abstract

A male patient with flu-like symptoms and tomography and laboratory diagnosis of severe acute respiratory syndrome. He developed acute cardiac dysfunction during admission and was submitted to a cardiac magnetic resonance imaging examination, which confirmed acute myocarditis, indicating cardiac involvement by coronavirus disease 2019. A review and discussion about coronavirus disease 2019-related cardiac manifestations are reported, focusing on the imaging findings to make diagnosis.

## INTRODUCTION

Since March 2020, we have been facing a pandemic due to the novel coronavirus (severe acute respiratory syndrome coronavirus 2 – SARS-CoV-2), whose initial cases emerged in the city of Wuhan, in the province of Hubei, China. ^( 1 - 4 )^

The clinical presentation spectrum is wide, from asymptomatic patients to critically ill cases. Most pulmonary infections are mild, but severe and critical cases have been described, especially in the elderly, developing with dyspnea, hypoxia, major lung involvement in imaging, respiratory failure, shock and multiple organ failure. ^( 5 )^

Chest computed tomography (CT) can help to diagnose the disease, mainly in the current pandemic scenario, in which real-time polymerase chain reaction (RT-PCR) results from nasal and oropharyngeal swabs can take a few days, although its use as a screening method is not recommended. The most frequently observed CT findings in cases of disease caused by SARS-CoV-2 are ground-glass opacities and consolidations in lungs, with a predominantly peripheral distribution, sometimes associated with fine reticulate (forming the so-called crazy-paving pattern), vascular thickening and inverted halo signal. Central parenchyma involvement, nodules, cavities, pleural effusion or lymph node enlargement are not frequently observed. ^( 6 , 7 )^

Cases of heart involvement by the coronavirus 2019 disease (COVID-19), developing with acute myocarditis have also been described, mainly in severe cases. ^( 2 , 8 )^ Chest CT, however, is limited in terms of heart assessment. ^( 9 )^ Thus, these patients with clinically suspected COVID-19 myocarditis have been assessed by other imaging methods, such as echocardiography and cardiac magnet resonance imaging (CMR). ^( 10 )^

We describe the case of a patient diagnosed with SARS-CoV-2 infection and cardiac involvement.

## CASE REPORT

**Figure 1:**
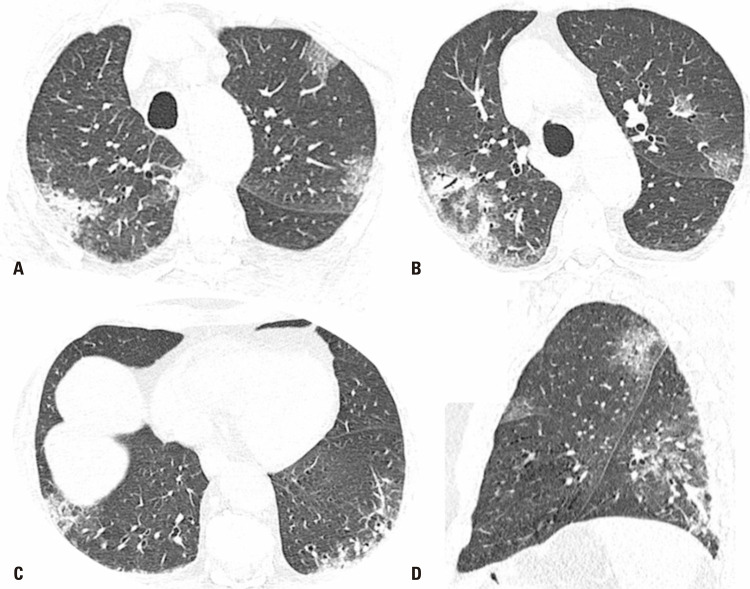
Chest computed tomography. Axial (A, B and C) and sagittal (D) images of chest computer tomography performed during inpatient stay, showing several ground-glass opacities and consolidation foci in all pulmonary lobes, compatible with infectious disease, suggestive of COVID-19 in appropriate clinical environment

**Figure 2:**
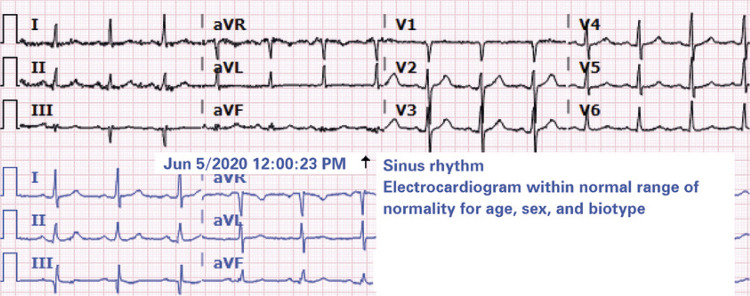
Electrocardiogram within normal range of normality for age, sex, and biotype

**Figure 3:**
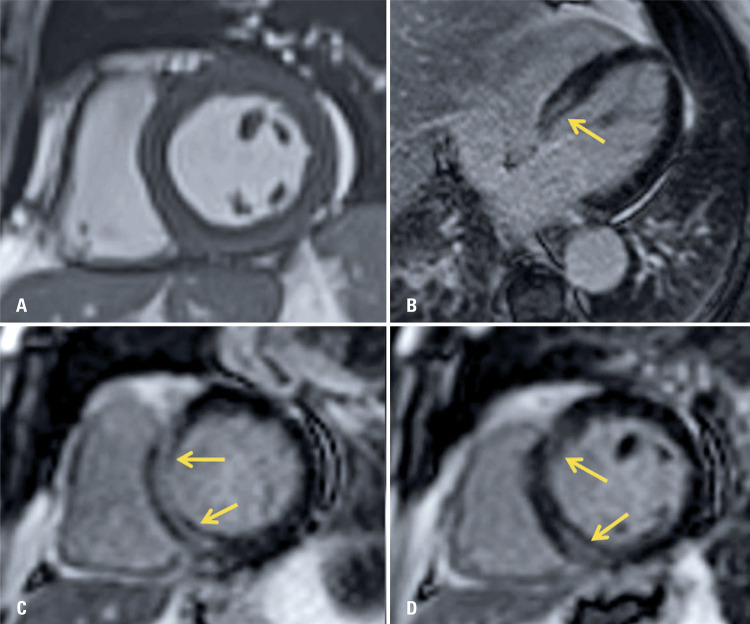
Cardiac magnet resonance imaging images on short axis. (A) and late myocardial enhancement sequences on the long axis of the four chambers (B) and short axis; (C and D) showing areas of late enhancement with non-ischemic pattern on the septal base wall of the left ventricle (arrows), findings that, in the clinical scenario, suggest inflammatory/infectious process (myocarditis)

An 81-year-old male patient came to the emergency room presenting fever (38.8°C), dyspnea and a 91% oxygen saturation at home, one day before. Real-time polymerase chain reaction SARS-CoV-2 identification was positive on a nasal and oropharyngeal swab sample. Polymerase chain reaction panel for respiratory pathogens was performed and did not reveal signs of coinfection. Given the clinical presentation and risk factors for progressing to a severe case, such as age, hypertension, and history of ischemic stroke, management chosen was admission and performing chest CT. The CT study revealed ( Figure 1 ) small round ground-glass opacities, with multifocal distribution on both lungs, more evident on the left peri-hilar region, which corroborated the possibility of COVID-19 among differential diagnoses. Admission lab tests revealed high troponin T (33pg/mL; normal if <5pg/mL). An electrocardiogram was then performed ( Figure 2 ), but did not show signs of ischemia, and the echocardiogram presented a reduction in the ejection fraction in relation to a previous study performed 7 months earlier (from 45% to 35%). The diagnosis of myocarditis of viral etiology by SARS-CoV-2 was considered, and an CMR requested for confirmation one day after hospital admission. The CMR revealed the presence of late enhancement areas with an ischemic pattern on the left ventricle base septum wall, along with pronounced diffuse hypokinesia, and global systolic function involvement, confirming the presumptive diagnosis of myocarditis related to the new coronavirus ( Figure 3 ).

The patient was treated with antibiotics, steroids and hemodynamic monitoring with increasing improvement of clinical symptoms and progressively normal laboratory tests, after 3 weeks of therapy. He was discharged on anticoagulant treatment (40mg/day), for 5 more days.

## DISCUSSION

COVID-19 cases with cardiac involvement, developing acute myocarditis have been described. ^( 2 , 8 )^ Heart failure has been appointed as one of the sources of secondary complications in these patients. ^( 8 )^

The analysis of 44,672 confirmed cases of COVID-19 in Wuhan pointed out cardiovascular complications, such as myocarditis (10% of cases), myocardial injury (20%), arrhythmias, (16%) and heart failure and shock (5%). ^( 11 - 13 )^

The mechanisms of cardiac involvement observed in COVID-19 are possibly due to direct viral infection to the myocardium or by the indirect toxicity caused by the systemic infection, and can trigger vasculitis or hypersensitivity reaction. ^( 14 )^

Inciardi et al., ^( 15 )^ reported a patient with COVID-19 and myocarditis diagnosed by CMR, who presented increased troponin, changes in segmental contractions and left ventricular dysfunction on the echocardiogram. The patient was treated with inotropic support, having improved clinically as of the first week after initiation of treatment. Another more severe case was reported by Hu et al., ^( 2 )^ who described a patient with the diagnosis of a fulminant myocarditis, along with diffuse myocardial edema and major ventricular dysfunction. The patient received hemodynamic support, steroids and human immunoglobulin, having completely recovered ventricular function and attained normal myocardial lesion markers after 3 weeks.

In the scenario of the SARS-CoV-2 pandemic, it is important to consider the hypothesis of cardiac involvement, mainly in patients with abrupt deterioration of symptoms despite respiratory support measures, those with unexplained increase in myocardial necrosis markers and in patients with a new dysfunction documented by echocardiography. In face of such a possibility, CMR can be used to search for signs compatible with myocarditis, such as the presence of non-ischemic late enhancement pattern.

Moreover, in suspected arrhythmia and/or myocarditis, lung fields should be carefully assessed, even by CMR, given respiratory asymptomatic or oligosymptomatic individuals can be infected by the new coronavirus, and suspicion can be considered as of this exam. ^( 10 )^

## CONCLUSION

The infection by SARS-CoV-2 can present cardiac manifestations, such as acute myocarditis, and monitoring and follow-up of acute heart failure are required. Complementary tests such as echocardiogram and cardiac magnet resonance imaging can help diagnostic investigation. Control of progression is indispensable, given there is still no evidence in the literature on the late development of myocardial dysfunction in these patients.
