# Supplementary material for: COVID-19 myocarditis: a case report
Source: Einstein (Sao Paulo). 2020 Oct 20;18:eRC5876. doi: 10.31744/einstein_journal/2020RC5876 (PMC7575039; doi:10.31744/einstein_journal/2020RC5876)
Supplement: Translation

# Translation: Miocardite na COVID-19: um relato de caso

**Authors:** Patrícia Yokoo, Eduardo Kaiser Ururahy Nunes Fonseca, Roberto Sasdelli, Walther Yoshiharu Ishikawa, Murilo Marques Almeida Silva, Elaine Yanata, Rodrigo Caruso Chate, Antonio Carlos Bacelar Nunes, Marcelo Bettega, João Ricardo Cordeiro Fernandes, Flávio Tarasoutchi, Gilberto Szarf

**Affiliations:** 1 Brasil Hospital Israelita Albert Einstein, São Paulo, SP, Brasil .

**Keywords:** Coronavírus, COVID-19, Infecções por coronavírus, Miocardite, Cardiomiopatias, Doenças transmissíveis, Síndrome respiratória aguda grave, Pneumonia, Ecocardiografia, Tomografia computadorizada por raios X

## Abstract

Paciente do sexo masculino apresentando síndrome gripal aguda com diagnóstico tomográfico e laboratorial de infecção por síndrome respiratória aguda grave. Evoluiu com disfunção cardíaca aguda durante a internação, motivo pelo qual foi submetido à ressonância magnética cardíaca, que confirmou miocardite aguda, indicando acometimento cardíaco por COVID-19. Foram realizadas revisão e discussão sobre o acometimento cardíaco na COVID-19, com ênfase nos aspectos por imagem para o diagnóstico.

## INTRODUÇÃO

Desde março de 2020, vivemos uma pandemia decorrente do novo coronavírus (coronavírus da síndrome respiratória aguda grave 2 – SARS-CoV-2), cujos primeiros casos surgiram na cidade de Wuhan, província de Hubei, na China.^( 1 - 4 )^

O espectro da apresentação clínica é amplo, desde pacientes assintomáticos até casos de doença crítica. A maior parte das infecções pulmonares é leve, porém formas graves ou críticas são descritas, especialmente em idosos, podendo cursar com dispneia, hipóxia, grande envolvimento pulmonar nos estudos por imagem, insuficiência respiratória, choque e insuficiência de múltiplos órgãos.^( 5 )^

A tomografia computadorizada (TC) do tórax pode ajudar no diagnóstico dessa doença, principalmente no contexto atual da pandemia, no qual os resultados do teste de reação em cadeia da polimerase em tempo real (RT-PCR) obtidos por *swab* nasal e de orofaringe podem demorar alguns dias, apesar de não ser recomendável seu uso como método de rastreamento. Os achados tomográficos mais frequentemente observados nos casos da doença desencadeada pelo SARS-CoV-2 são as opacidades pulmonares em vidro fosco e consolidações, com distribuição predominantemente periférica, por vezes associadas a reticulado fino (configurando o chamado padrão de pavimentação em mosaico), espessamento vascular e sinal do halo invertido. São infrequentes o acometimento central do parênquima, nódulos, cavidades, derrame pleural ou linfonodomegalias.^( 6 , 7 )^

Casos de acometimento cardíaco pela doença pelo coronavírus 2019 (COVID-19) cursando com miocardite aguda também vem sendo descritos, principalmente em doentes graves.^( 2 , 8 )^A TC do tórax, no entanto, é limitada na avaliação do coração.^( 9 )^Assim, esses pacientes com suspeita clínica de miocardite na COVID-19 são avaliados por outros métodos de imagem, como o ecocardiograma e a ressonância magnética cardíaca (RMC).^( 10 )^

Decrevemos o caso de um paciente diagnosticado com a infecção pelo SARS-CoV-2 e envolvimento cardíaco.

## RELATO DE CASO

Paciente do sexo masculino, 81 anos de idade, procurou o serviço de emergência com quadro de febre (38,8°C), dispneia e saturação de oxigênio de 91% em domicílio, há 1 dia. A identificação do SARS-CoV-2 na RT-PCR foi positiva em amostra obtida por *swab* nasal e da orofaringe. Também foi realizada PCR para painel de patógenos respiratórios, que não demonstrou sinais de coinfecção. Diante da clínica e dos fatores de risco de evolução para quadro grave, como idade, hipertensão arterial sistêmica e antecedente de acidente vascular cerebral isquêmico, optou-se pela internação hospitalar e pela realização de TC de tórax. O estudo tomográfico de tórax ( Figura 1 ) demonstrou pequenas opacidades em vidro fosco arredondadas, com distribuição multifocal em ambos os pulmões, sendo a mais evidente na região peri-hilar esquerda, o que corroborou a possibilidade de COVID-19 entre os diagnósticos diferenciais. Seus exames laboratoriais de entrada demonstraram aumento da troponina T (33pg/mL; normal se <5pg/mL). Foi, então, realizado eletrocardiograma ( Figura 2 ), que não demonstrava sinais de isquemia e ecocardiograma, com redução da fração de ejeção em relação ao estudo prévio realizado há 7 meses (de 45% para 35%). O diagnóstico de miocardite de etiologia viral pelo SARS-CoV-2 foi considerado, sendo solicitada RMC para sua confirmação 1 dia depois da internação hospitalar. A RMC demonstrou presença de áreas de realce tardio de padrão não isquêmico na parede septal basal do ventrículo esquerdo, acompanhadas de acentuada hipocinesia difusa, com comprometimento da função sistólica global, confirmando a hipótese diagnóstica de miocardite relacionada ao novo coronavírus ( Figura 3 ).

**Figura 1:** Tomografia computadorizada do tórax. Imagens axiais (A, B e C) e sagital (D) da tomografia computadorizada do tórax realizadas durante a internação, mostrando várias opacidades em vidro fosco e focos de consolidação em todos os lobos pulmonares, compatíveis com doença infecciosa, sugestivas de COVID-19 em ambiente clínico adequado

**Figura 2:** Eletrocardiograma dentro dos limites da normalidade para idade, sexo e biótipo

**Figura 3:** Imagens de ressonância magnética cardíaca na cinerressonância magnética no eixo curto. (A) e sequências de realce miocárdico tardio no eixo longo quatro câmaras; (B) e eixo curto; (C e D) mostrando áreas de realce tardio de padrão não isquêmico na parede septal basal do ventrículo esquerdo (setas), achados que, no contexto clínico, sugerem processo inflamatório/infeccioso (miocardite)

Foi tratado com antibioticoterapia, corticoterapia e monitoramento hemodinâmico com melhora progressiva dos sintomas clínicos e normalização dos exames laboratoriais após 3 semanas de tratamento. Recebeu alta em uso de anticoagulante (40mg/dia), por mais 5 dias.

## DISCUSSÃO

Casos de acometimento cardíaco pela COVID-19 cursando com miocardite aguda vêm sendo descritos.^( 2 , 8 )^A insuficiência cardíaca tem sido apontada como uma das fontes de complicação secundária nesses pacientes.^( 8 )^

A análise de 44.672 casos confirmados de COVID-19 em Wuhan evidenciou complicações cardiovasculares, como miocardite (10% dos casos), injúria miocárdica (20%), arritmias (16%) e insuficiência cardíaca e choque (5%).^( 11 - 13 )^

Os mecanismos de envolvimento cardíaco observados na COVID-19 são possivelmente atribuídos pela infecção viral direta ao miocárdio ou pela toxicidade indireta ocasionada pela infecção sistêmica, podendo desencadear vasculite ou reação de hipersensibilidade.^( 14 )^

Inciardi et al.,^( 15 )^descrevem um relato de uma paciente com COVID-19 e miocardite diagnosticada na ressonância magnética, que apresentou aumento de troponina, alterações de contração segmentar e disfunção ventricular esquerda ao ecocardiograma. Foi tratada com suporte inotrópico, apresentando melhora clínica a partir de 1 semana após instituição do tratamento. Outro caso mais grave foi relatado por Hu et al.,^( 2 )^que descreveu um paciente com diagnóstico de miocardite fulminante, o qual apresentou edema miocárdico difuso e disfunção ventricular importante. Foi tratado com suporte hemodinâmico, corticoide e imunoglobulina humana, com recuperação completa da função ventricular e normalização dos marcadores de lesão miocárdica após 3 semanas.

No contexto de pandemia pelo SARS-CoV-2, é importante que a hipótese de acometimento cardíaco seja considerada, principalmente naqueles pacientes com piora abrupta da sintomatologia a despeito de medidas de suporte respiratório, nos com aumento inexplicado de marcadores de necrose miocárdica e em pacientes com disfunção nova documentada pela ecocardiografia. Diante dessa possibilidade, a RMC pode ser utilizada para a pesquisa dos sinais compatíveis com miocardite, como presença de realce tardio de padrão não isquêmico.

Além disso, nos casos de suspeita de arritmia e/ou miocardite, devem-se avaliar cuidadosamente os campos pulmonares, mesmo pela RMC, uma vez que indivíduos assintomáticos ou oligossintomáticos respiratórios podem estar infectados pelo novo coronavírus, e essa suspeita pode ser considerada a partir desse exame.^( 10 )^

## CONCLUSÃO

A infecção pelo SARS-CoV-2 pode apresentar manifestações cardíacas, como miocardite aguda, sendo necessários monitoramento e acompanhamento de insuficiência cardíaca aguda. Exames complementares, como ecocardiograma e ressonância magnética cardíaca, podem auxiliar na investigação diagnóstica. O controle evolutivo desses pacientes é imprescindível, visto que ainda não há evidências na literatura sobre a evolução tardia da disfunção miocárdica nesses pacientes.
